# Next-Generation Natural Hydrogels in Oral Tissue Engineering

**DOI:** 10.3390/pharmaceutics17101256

**Published:** 2025-09-25

**Authors:** Mariana Chelu, Monica Popa, José María Calderón Moreno

**Affiliations:** “Ilie Murgulescu” Institute of Physical Chemistry, 202 Splaiul Independentei, 060021 Bucharest, Romania; mchelu@icf.ro

**Keywords:** natural polymer hydrogels, oral health, periodontal therapy, biocompatibility, antimicrobial properties, extracellular matrix, regenerative dentistry, alveolar bone regeneration

## Abstract

Hydrogels have emerged as promising biomaterials for oral tissue regeneration thanks to their high-water content, excellent biocompatibility, and ability to mimic native tissue environments. These versatile materials can be tailored to support cell adhesion, proliferation, and differentiation, making them suitable for repairing both soft and hard oral tissues. When engineered from natural polymers and enriched with bioactive agents, hydrogels offer enhanced regenerative potential. Biopolymer-based hydrogels, derived from materials such as chitosan, alginate, collagen, hyaluronic acid, and gelatin, are particularly attractive due to their biodegradability, bioactivity, and structural similarity to the extracellular matrix, creating an optimal microenvironment for cell growth and tissue remodeling. Recent innovations have transformed these systems into multifunctional platforms capable of supporting targeted regeneration of periodontal tissues, alveolar bone, oral mucosa, dental pulp, and dentin. Integration of bioactive molecules, particularly essential oils, bio-derived constituents, cells, or growth factors, has introduced intrinsic antimicrobial, anti-inflammatory, and antioxidant functionalities, addressing the dual challenge of promoting tissue regeneration while at the same time attenuating microbial contamination in the oral environment. This review explores the design strategies, material selection, functional properties, and biomedical applications in periodontal therapy, guided tissue regeneration, and implant integration of natural polymer-based hydrogels enriched with bioactive factors, highlighting their role in promoting oral tissue regeneration. In addition, we discuss current challenges related to mechanical stability, degradation rates, and clinical translation, while highlighting future directions for optimizing these next-generation bioactive hydrogel systems in regenerative dentistry.

## 1. Introduction

Oral health is a fundamental component of overall health and quality of life, affecting essential functions such as nutrition, speech, esthetics and social interactions [1]. However, the oral cavity is constantly exposed to mechanical forces, microbial colonization and environmental challenges that can lead to conditions such as periodontitis, dental caries, and oral mucosal defects [2]. Conventional treatments, such as surgery, grafting, and prosthetic replacement, mainly focus on halting disease progression or replacing lost structures, rather than achieving true biological regeneration [3].

Tissue engineering has emerged as a promising transformative field in modern dentistry, with a multidisciplinary approach that combines biomaterials, cells and signaling molecules for the functional and structural regeneration of damaged tissues [4]. In dentistry, oral tissue engineering has the potential to restore various complex structures, including gingival tissues, periodontal ligament, alveolar bone, and dental pulp. Among the main components of tissue engineering, biomaterial scaffolds are crucial for providing a three-dimensional (3D) framework that supports cellular activities and tissue formation [5].

Natural hydrogels (NHGs), derived from biopolymers such as collagen, gelatin, chitosan, alginate, and hyaluronic acid, have attracted significant attention due to their structural similarity to the extracellular matrix (ECM), high water content, excellent biocompatibility, and bioactive properties [6,7]. Their hydrated and porous networks allow for efficient transport of nutrients and oxygen, creating a favorable microenvironment for tissue regeneration. Offering distinct advantages over their synthetic counterparts, these hydrogels not only facilitate cell adhesion, proliferation, and differentiation, but also allow for the controlled delivery of bioactive agents, making them ideal candidates for oral tissue regeneration applications.

**Figure 1:**
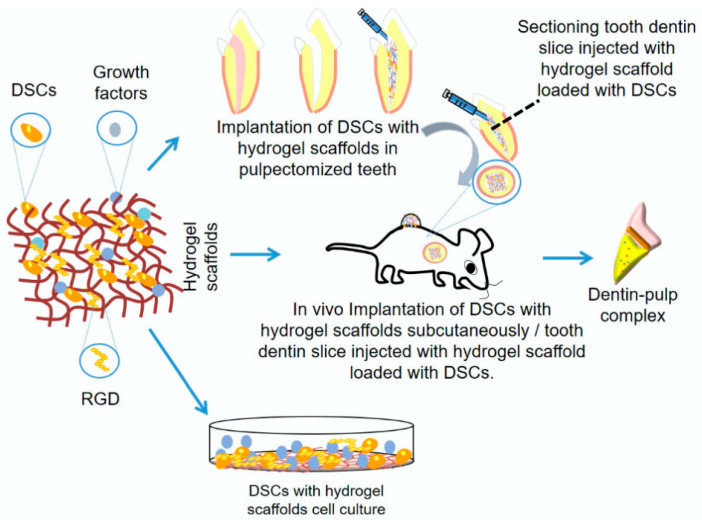
Graphical representation of different methods involving hydrogel scaffolds for dentin-pulp complex regeneration [9].

In recent years, biofunctionalization by the incorporation of essential oils (EOs), and some bioactive components such as growth factors (fibroblast growth factor, transforming growth factor, bone morphogenetic protein, transforming growth factor and vascular endothelial growth factor), cells (stem cells, chondrocytes, macrophages and osteoblasts), and nanoparticles into NHGs has opened new possibilities for multifunctional scaffolds in oral tissue engineering [8]. EOs, with their antimicrobial, anti-inflammatory, and antioxidant properties, can enhance the therapeutic potential of hydrogels by reducing the risk of infection and modulating local inflammation. This integration creates bioactive scaffolds, with a suitable degree of flexibility and elasticity, capable of not only supporting tissue regeneration but also protecting against microbial challenges of the oral environment, thus bridging the gap between structural support and active biological protection (Figure 1) [9,10].

**Figure 2:**
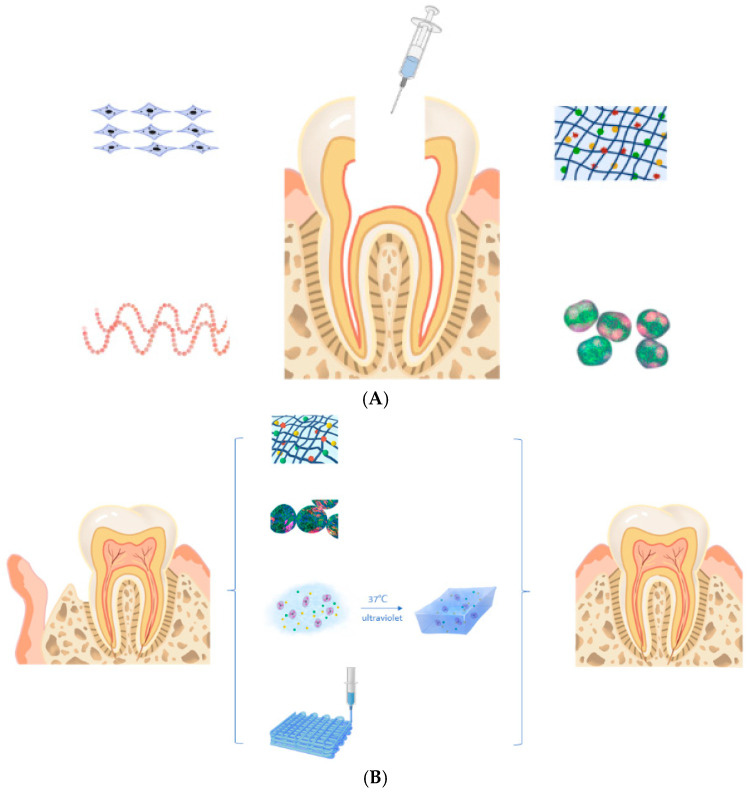
Application of hydrogel in (**A**) pulp and (**B**) periodontal tissue regeneration [11].

This review explores the recent advances, challenges and future prospects of the use of NHGs in oral tissue engineering. It highlights their applications in periodontal regeneration, bone repair, dental pulp regeneration, and soft tissue healing, emphasizing their role in advancing regenerative dentistry toward clinical translation (Figure 2) [11].

### 1.1. Importance of Oral Tissue Engineering

Oral tissue engineering is of critical importance in addressing the regenerative demands of the oral and maxillofacial region, where a variety of hard and soft tissues work together. Diseases such as periodontitis, a chronic inflammatory condition that leads to the destruction of tooth-supporting structures, affect almost half of the global adult population and are a major cause of tooth loss [12]. In addition, dental caries, trauma, tumors, and congenital defects often lead to significant loss of oral tissue, severely affecting both esthetics (with associated psychological repercussions) and function [13].

Beyond local consequences, oral diseases are increasingly recognized for their association with systemic conditions, including cardiovascular disease, diabetes, and respiratory disorders, underscoring the importance of effective oral tissue regeneration strategies [14]. Traditional treatments, such as bone grafts and barrier membranes, offer some regenerative potential, but are limited by donor site morbidity, immunogenic responses, and unpredictable outcomes [15].

**Figure 3:**
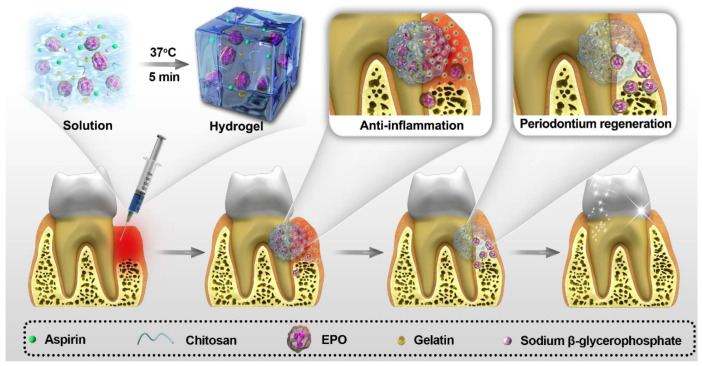
The use of hydrogel-based complexes in periodontal therapy [17].

Oral tissue engineering aims to overcome these challenges by integrating bioactive materials such as hydrogels incorporating bio-derived compounds, stem cells, or growth factors, to recreate the native tissue environment and promote functional regeneration [16]. NHGs serve as versatile scaffolds that mimic the ECM, support cellular processes, and facilitate the delivery of therapeutic agents, making them an integral part of regenerative approaches in dentistry (Figure 3) [17].

The ability to biologically restore the structure and function of lost or damaged oral tissues not only improves oral health but also improves patients’ quality of life and overall systemic well-being. With advances in hydrogels science and biofabrication technologies, oral tissue engineering is poised to transform future dental therapies, moving towards personalized, minimally invasive, and highly effective regenerative solutions [18].

The importance of oral tissue engineering lies in its potential in the following [19]:

- Restore the structure and function of native tissue, rather than replacing it with inert materials;
- Promote natural healing and regeneration through the administration of cells, growth factors, and supportive scaffolds;
- Reduce the need for autografts or allografts, minimizing donor site morbidity and the risks of immune rejection;
- Enable personalized treatment strategies, particularly due to advances in 3D bioprinting and patient-specific scaffold design.

### 1.2. Challenges in Oral Tissue Regeneration

Oral tissue regeneration presents unique biological and clinical challenges due to the complex anatomy, dynamic environment, and diverse tissue types of the oral cavity. The oral environment is constantly subjected to mechanical forces from mastication, salivary flow, microbial biofilms, and pH fluctuations, all of which can compromise the stability and functionality of regenerative materials and cell therapies [20].

One of the main challenges in oral tissue engineering is recreating the hierarchical structure of oral tissues, particularly in the periodontium, which consists of alveolar bone, periodontal ligament (PDL), cementum, and gingiva, each with distinct biological functions and regenerative requirements [21].

Vascularization is another significant barrier, especially in the case of large or complex tissue defects. Successful tissue regeneration relies on the formation of a functional vascular network to ensure adequate nutrient and oxygen supply to transplanted cells and newly formed tissues. However, hydrogels and other scaffolds often lack intrinsic vasculogenic properties, delaying or limiting regeneration [22].

Furthermore, many commonly used materials fail to provide the mechanical strength required to withstand the functional stresses in the oral cavity. NHGs often have poor mechanical stability, limiting their use in load-bearing applications such as alveolar bone regeneration [23].

The host immune response adds another layer of complexity. Biomaterials can trigger inflammatory reactions or fail to integrate with host tissues, leading to fibrosis or scaffold rejection. Striking a balance between immunomodulation and regenerative support is essential for long-term success [24].

From a clinical perspective, translational challenges persist, including scalability, reproducibility, regulatory hurdles, and cost-effectiveness. The customization of scaffolds and cell-based therapies for individual patients adds to these complexities, slowing their adoption into routine dental practice [25]. Overcoming these challenges requires continued innovation in biomaterial design, biofabrication technologies, and interdisciplinary collaborations to develop clinically viable solutions that mimic the native tissue environment and promote functional regeneration.

## 2. Natural Hydrogels

### 2.1. Composition, Properties and Manufacturing Methods

In this chapter we will provide a brief overview of the characteristics of NHGs applied in regenerative medicine and especially in oral tissue engineering.

Hydrogels are three-dimensional network structures made from synthetic and/or natural polymers, capable of absorbing and retaining large amounts of water [26,27]. These swollen, viscoelastic polymeric networks share many physical properties with natural tissues [28]. Their framework is created through crosslinking, which can occur via covalent bonds or noncovalent interactions, and can be tailored to meet specific application needs [29,30].

The exceptional water-holding capacity of hydrogels stems from their hydrophilic nature, with the extent of water absorption influenced by factors such as network structure, crosslink density, solution composition, and the synthesis method employed [31].

Hydrogels can be classified in several ways: by origin, as natural or synthetic, or by crosslinking method, as physically or chemically crosslinked [32,33]. Their high versatility comes from the fact that both their chemical composition and physical properties can be adjusted, enabling the design of gels suited to a wide range of uses [34]. The swollen 3D network also allows the diffusion of molecules, further expanding their functional potential [35].

NHGs, include collagen, silk fibroin, hyaluronic acid, chitosan, alginate and hydrogels derived from decellularized tissues [36,37]. Their unique properties include biocompatibility, biodegradability, low cytotoxicity, the possibility to tailor the hydrogel into an injectable gel, and their similarity to physiological environment. However, NHGs do have some limitations; for example, they do not have strong mechanical properties and are not easily controllable due to their batch-to-batch variation. For these reasons, NHGs are often combined with synthetic ones, creating composite polymers, and are still widely experimented [38,39].

Hydrogels, in general, have been extensively studied for different biomedical applications, which include drug delivery systems, 3D cultures, tissue implant, tissue regeneration, and use in healthcare products [40,41]. Every different type of hydrogel can be tailored to suit the application it is designed for; therefore, hydrogels will be made using different techniques to give them the necessary chemical and physical properties [42,43].

Hydrogels are generally non-toxic and well-tolerated by biological systems due to their high water content, soft texture, and similarity to natural tissues [44,45]. They minimize immune responses and can be engineered to enhance cell adhesion or resist biofouling, depending on the application [46,47].

Biodegradable hydrogels can break down into non-toxic byproducts through hydrolysis, enzymatic action, or other physiological processes. The degradation rate can be controlled by polymer type, crosslinking density, and environmental conditions, allowing temporary or long-term functionality as needed [48].

In terms of mechanical properties, hydrogels exhibit soft, elastic and flexible behavior, often resembling natural tissues [49]. Their strength, toughness and elasticity depend on the polymer composition, crosslink density and water content. They can range from weak, gelatinous gels to hard, load-bearing materials.

Similarly, hydrogels exhibit viscoelastic properties, exhibiting both solid (elastic) and liquid (viscous) responses to deformation [50]. Their rheology is influenced by the network structure, polymer concentration, temperature and swelling state, which determine their deformation, flow and recovery characteristics [51].

Hydrogels can be designed to actively interact with biological systems by incorporating bioactive molecules, peptides or growth factors. This allows them to support tissue regeneration, promote healing, or deliver therapeutic agents [52,53].

Depending on their composition and surface properties, hydrogels can promote or inhibit cell adhesion, proliferation, and differentiation. Functionalization with cell recognition motifs (e.g., RGD peptides) enhances cell attachment, while inert surfaces can minimize unwanted cell interactions [54].

NHGs are derived from biological materials such as (i) polysaccharides (e.g., alginate from brown algae, agar and carrageenan from red algae, chitosan from crustacean shells, cellulose from plants) and (ii) proteins (e.g., gelatin from collagen, fibrin from blood plasma, silk fibroin from silkworms, and elastin from connective tissues). These sources provide inherent biocompatibility and biodegradability, making them suitable for biomedical and food applications [55,56].

The hydrogels from natural polysaccharides such as alginate, chitosan, agarose, hyaluronic acid, and cellulose derivatives are biocompatible, biodegradable, and often non-toxic, making them ideal for biomedical and pharmaceutical applications. Their properties, such as gelation behavior, mechanical strength, and bioactivity, can be tuned by chemical modification or blending with other polymers [57].

Composite hydrogels are formed by incorporating fillers such as nanoparticles, fibers, or other polymers into a hydrogel matrix to enhance mechanical strength, stability, or functionality [58,59]. Hybrid hydrogels combine natural and synthetic polymers, leveraging the biocompatibility of natural components with the tunable properties of synthetic ones. Both types offer improved performance for applications in tissue engineering, drug delivery, and wound healing [60].

Hydrogels are formed by crosslinking, which connects polymer chains to create a 3D network [61].

Physical crosslinking involves noncovalent interactions, such as hydrogen bonds, ionic interactions, hydrophobic interactions, or crystallization. These hydrogels are typically reversible and responsive to environmental changes [62].

Chemical crosslinking induces the formation of covalent bonds between polymer chains using chemical agents or UV light. These hydrogels are typically stronger and more stable than physically crosslinked ones [63].

Enzymatic crosslinking uses specific enzymes to catalyze the formation of covalent bonds between polymer chains, creating a stable 3D hydrogel network. It is a facile, highly specific method that occurs under physiological conditions, making it ideal for biomedical applications such as tissue engineering and drug delivery. It also allows for precise control over the gelation rate and properties of the hydrogel [64].

### 2.2. Advantages and Constraints of NHGs in Dentistry

The advancement of innovative materials remains essential in oral tissue engineering [65]. In this regard, NHGs have attracted significant attention in dentistry, especially in the field of oral tissue engineering, due to their biocompatibility, and bioactivity [66]. These materials provide a versatile and supportive environment for the attachment, proliferation, differentiation and regeneration of cellular tissues. The main advantages of using NHGs in oral tissue engineering are determined by several characteristics:

(i) Biocompatibility. NHGs are derived from biological sources such as collagen, gelatin, chitosan, alginate, hyaluronic acid, and fibrin, which are inherently compatible with living tissues. They present a minimized immune response and in general, these materials are well tolerated by the body, reducing the risk of inflammation or rejection. In addition, it is a cell-friendly environment, supporting the growth and function of oral cells such as fibroblasts, keratinocytes, osteoblasts, and dental pulp stem cells (DPSCs) [67].
(ii) Biomimetics of the ECM. NHGs closely mimic the structure and composition of the ECM found in soft and hard oral tissues. Their 3D porous architecture allows for the diffusion of nutrients and oxygen, promoting cell survival. Many NHGs retain bioactive motifs within the structure of biopolymers and constituents, which can interact directly with cell receptors, enhancing cell adhesion and signaling [68].
(iii) Biodegradability and controlled degradation. Another advantage of NHGs is their ability to degrade in a controlled and predictable manner, matching the physiological rhythm of tissue regeneration. Degradation rates can be adjusted to allow the scaffold to gradually disappear as new tissue forms, reducing the need for surgical removal. HGs degradation products are usually non-toxic and can be metabolized or excreted naturally [69].
(iv) Improved cell adhesion and proliferation. NGHs often contain cell adhesion molecules, such as arginine–glycine–aspartic acid (RGD) sequences, which promote better cell-skeleton interaction. At the same time, improved cell adhesion facilitates proliferation and differentiation, accelerating healing and regeneration. From epithelial to mesenchymal stem cells, NHGs can support various types of oral cells [70].
(v) Injectable and thermoresponsive properties. Many NHGs, such as gelatin or chitosan-based formulations, can be administered in a minimally invasive manner as injectable gels. They can transition from liquid to gel at body temperature, perfectly filling irregular defects. Not to be overlooked, this property is particularly useful for applications in delicate oral tissues or in areas that are difficult to access [71,72].
(vi) Versatility in applications. NHGs are adaptable to a wide range of applications in dental tissue engineering, including periodontal regeneration (supporting the regeneration of cementum, periodontal ligament and alveolar bone) and pulp and dentin regeneration (as carriers for DPSC-derived stem cells and growth factors to restore pulp vitality). Also, HGs are tunable for bone grafts (supporting mineralization and osteogenesis of maxillofacial and jaw bone defects) and oral mucosa repair (helping with re-epithelialization in cases of trauma or ulceration) [73].
(vii) Potential for delivery of growth factors and drugs. HGs can act as carriers for bioactive molecules, including growth factors (e.g., BMPs, VEGF, TGF-β), antimicrobial agents, or anti-inflammatory drugs. They can be engineered to release these agents in a controlled and sustained manner, improving local therapeutic outcomes. By delivering bioactive factors, enhanced tissue regeneration is supported [74].
(viii) Reduced risk of chronic inflammation and fibrosis. Compared to synthetic materials, NHGs are less likely to induce chronic inflammatory responses or fibrosis, both of which can hinder the long-term success of oral implants or grafts [75].
(ix) Eco-friendly and sustainable materials. Derived from natural sources, these NHGs are often renewable, eco-friendly, and cost-effective, which is beneficial for sustainable clinical practices and commercial production [76].

However, there are some disadvantages and challenges associated with the use of NHGs in dentistry. Here are a few:

(i) Weak Structural Integrity and Wear: NHGs often lack the mechanical strength and durability required for load-bearing applications in dentistry. For example, they might not withstand the forces exerted during chewing or other oral functions. Over time, hydrogels can degrade under mechanical stress, leading to wear and tear, which reduces their long-term effectiveness as dental materials.
(ii) Inconsistent Composition and Source Variability: NHGs, derived from biological sources like collagen, alginate, or chitosan, can have variable properties depending on the source and method of extraction. This can result in inconsistent performance across different batches. The availability and quality of natural materials can vary depending on environmental conditions and sourcing, which can also lead to supply chain issues or fluctuations in prices.
(iii) Variable Swelling Behavior: NHGs may absorb water differently based on environmental factors, which can influence their stability and performance in the oral environment.
(iv) Lack of Control Over Premature Degradation: While biodegradability is generally considered a positive feature, the rate of degradation can sometimes be too rapid for certain applications in dentistry. If the hydrogel degrades before the intended therapeutic or restorative effect is achieved, the treatment may fail. It can be challenging to precisely control the rate of biodegradation, leading to inconsistent outcomes in dental procedures.
(v) Microbial Colonization and Infection Risk: NHGs, particularly those derived from polysaccharides and proteins, can be more susceptible to microbial colonization and infection compared to synthetic materials. This is a significant concern in the oral cavity, where the presence of bacteria is prevalent. If the hydrogel degrades prematurely or has poor antimicrobial properties, it may increase the risk of secondary infections, particularly in periodontal treatments or wound healing after surgery.
(vi) Sensitivity to Heat and Chemicals and Limited Shelf Life: NHGs can be sensitive to heat and chemical sterilization methods. Traditional sterilization processes might degrade or alter their properties, compromising their effectiveness as dental materials. Due to their natural origins, some hydrogels may have a shorter shelf life compared to synthetic materials, which can be a logistical issue for dental practices.
(vii) Production and Processing Costs: NHGs may be more expensive to produce, especially if they are derived from complex or rare biological sources. This could make them less cost-effective compared to synthetic alternatives. The fabrication of NHGs for dental applications can involve complex processes to ensure uniformity and desired properties. This adds to the difficulty and cost of incorporating them into dental treatments.
(viii) Unknown Long-Term Effects: While NHGs show promise in short-term studies, there is often a lack of comprehensive long-term data regarding their stability, biocompatibility, and potential adverse effects when used in dentistry. This makes clinicians hesitant to adopt them for more permanent dental treatments.
(ix) Compatibility Issues: When used in conjunction with other materials, such as dental composites, adhesives, or metals, NHGs may not always exhibit optimal compatibility. This could affect the longevity and performance of restorative procedures like fillings, crowns, or implants. Table 1 outlines the main benefits and limitations of natural and synthetic hydrogels when applied in dental treatments.

Although NHGs are inherently more biocompatible than synthetic alternatives, none of them uniquely offer the ideal combination of mechanical integrity, bioactivity, and controlled degradation. This has led to strategies involving improved crosslinking, the formation of composite hydrogels, or functionalization with other bioactive components. Table 2 outlines the benefits and drawbacks of naturally derived hydrogels.

Functionalization can be achieved with various bioactive agents, including the following:

(A) Essential oils (EOs), e.g., eugenol, tea tree oil, thymol, carvacrol [77]. The main advantages of their use are natural antimicrobial activity (important for periodontal areas prone to infection), anti-inflammatory and antioxidant properties, as well as cost-effectiveness and ease of integration into the hydrogel matrix. At the same time, there are also limitations to the incorporation of EOs, which are most often related to the following: volatility and instability over time; the hydrophobic nature of EOs that limits dispersion in hydrogels and the potential cytotoxicity at higher concentrations.

Thus, although promising for antimicrobial action, the integration of essential oils requires nanoencapsulation or emulsion stabilization to ensure controlled release and compatibility with hydrophilic matrices. This topic will be further developed in the next section.

(B) Nanoparticles (NP). Several types of NPs such as silver (Ag), gold (Au), zinc oxide (ZnO), mesoporous silica, bioactive glass, polymeric NPs can be incorporated into hydrogel matrices. Obviously, the main advantages they bring are demonstrated by the improvement of the antibacterial properties (especially Ag, ZnO) of NHGs, the ability to act as drug/growth factor carriers and the improvement of mechanical strength and bioactivity. However, there are also some limitations regarding the inclusion of NPs. These are mainly related to the potential toxicity depending on the type of particles and dose, and the challenges regarding the homogeneity of the dispersion of NPs in the hydrogel matrix. In addition, regarding long-term biocompatibility, data are limited, not many studies have been carried out in this regard. A significant improvement in the functionality of NHGs can be driven in the future, by optimizing the dose and by functionalization, which should ensure sustained release and avoid triggering chronic inflammation.

(C) Use of growth factors (GFs), such as BMP-2, VEGF, PDGF, FGF, and TGF-β. The main benefit is the direct stimulation of cell proliferation, migration, and differentiation, as well as the crucial importance for angiogenesis (VEGF), osteogenesis (BMP-2), and tissue remodeling. However, there are also several barriers that need to be overcome, such as short biological half-life, sensitivity to environmental degradation, and the risk of ectopic tissue formation if the composition design is not well regulated. This is because the administration of growth factors requires controlled release systems to prevent sudden release and achieve spatiotemporal control. Their high cost and regulatory hurdles restrict wide clinical use.

### 2.3. The Role of Essential Oils in NHGs for Oral Tissue Regeneration

The incorporation of organic active substances, such as EOs, into NHGs has attracted significant interest due to the potential synergistic effects that can enhance therapeutic efficacy for oral tissue regeneration [78,79].

EOs are volatile aromatic compounds extracted from plants and possess a wide range of bioactive properties, including antimicrobial, anti-inflammatory, antioxidant, and wound-healing effects [80]. Due to these characteristics, they have been used in traditional medicine since ancient times. When incorporated into NHGs, they can synergistically enhance the regenerative microenvironment in the oral cavity [81].

The main attributes of essential oil-loaded hydrogels in oral tissue regeneration encompass a wide range of functional, biological and therapeutic properties, such as the following:

*Targeted antimicrobial activity.* Oral tissues are constantly exposed to a diverse and dense microbial population. EOs, such as tea tree oil, clove oil, peppermint oil, melissa and cinnamon oil, exhibit broad-spectrum antibacterial, antifungal, and antiviral effects. Incorporating these oils into hydrogels can help suppress pathogenic microorganisms that contribute to periodontal disease, peri-implantitis, and post-surgical infections, thereby creating a cleaner environment conducive to tissue regeneration [82,83].

*Anti-inflammatory effects.* Chronic inflammation is a major barrier to successful regeneration in conditions such as periodontitis. Eos such as eucalyptus, lavender, ginger, and chamomile contain bioactive compounds (e.g., eugenol, linalool, α-bisabolol) that modulate inflammatory pathways. In hydrogels, these compounds can be delivered locally to the site of the defect, reducing the activity of pro-inflammatory cytokines and promoting a shift toward a pro-healing immune response [84].

*Antioxidant properties.* Oxidative stress in oral wounds, especially after surgical or periodontal procedures, can impair healing. Essential oils such as rosemary, oregano, and thyme are rich in phenolic compounds with strong antioxidant capacity, neutralizing reactive oxygen species (ROS) and protecting cells from oxidative damage [85,86].

Sustained administration via a hydrogel provides prolonged antioxidant action during the critical early stages of regeneration.

*Enhance wound healing and angiogenesis*. Certain EOs, such as lavender and rosehip, have been shown to promote fibroblast migration, collagen synthesis, and neovascularization. In NHGs (e.g., collagen, chitosan, or gelatin matrix), these oils can create a bioactive scaffold that not only provides structural support but also actively stimulates tissue remodeling and vascular growth [87,88].

*Synergy with the natural properties of the hydrogel*. NHGs such as chitosan, alginate, collagen and hyaluronic acid already offer biocompatibility and a structure that mimics the ECM. When EOs are incorporated into these hydrogels, they enhance the functionality of the scaffold by combining structural support with biochemical cues for healing [89]. For example: the combination of chitosan and tea tree oil shows antimicrobial and wound healing synergy for periodontal pockets [90]. Also, the incorporation of clove oil into gellan gum hydrogel leads to pain relief, having an anti-inflammatory and regenerative action in fighting bacterial infections linked to periodontitis [91].

A novel hydrogel containing tea tree oil encapsulated in solid lipid nanoparticles was investigated for the treatment of oropharyngeal candidiasis. It demonstrated a fungicidal effect applicable to the treatment of oropharyngeal candidiasis generated by a wide variety of Candida species [92].

*Controlled release and sustained bioactivity of bioactive compounds*. NHGs provide a hydrated, biocompatible matrix that allows encapsulation and can protect sensitive bioactive compounds from rapid evaporation and degradation, while facilitating controlled release. This ensures a sustained therapeutic effect directly at the defect site, reducing the need for repeated applications and minimizing systemic side effects [93]. EOs, rich in volatile and hydrophobic compounds, can interact with the polymer network of the hydrogel, influencing both its stability and release profile.

The chemical composition of an EO plays a key role in its release kinetics. For example, hydrophobic constituents can form interactions with similarly hydrophobic regions in a polymer matrix, such as chitosan, leading to a slower and more sustained release. Conversely, more polar components can diffuse more rapidly through the hydrogel network. By carefully selecting both the EO composition and the type of hydrogel, it is possible to modulate release rates, improve bioavailability, and target specific therapeutic outcomes, making these systems extremely versatile for applications in oral tissue engineering and drug delivery.

Potential applications in oral tissue regeneration of essential oils encapsulated in hydrogel:

- In periodontal regeneration, it can support infection control, modulate inflammation and promote the formation of new alveoli [81].
- In post-extraction alveoli healing, it leads to a reduction in the incidence of dry socket, accelerating soft tissue closure [94].
- It contributes to implant site preparation by reducing peri-implant microbial load and promotes osseointegration [95].
- Repair of mucosal defects in cases of oral ulcers or trauma, promoting epithelial coverage and comfort [96].

A comprehensive comparison presented in Table 3 highlights the distinct advantages and limitations of NHG systems in contrast to their synthetic and hybrid counterparts, in terms of composition and origin, biocompatibility and bioactivity, mechanical performance and stability, degradation and biodegradability, as well as processability and functionalization.

## 3. Overview of Oral Tissues and Regeneration

**Figure 4:**
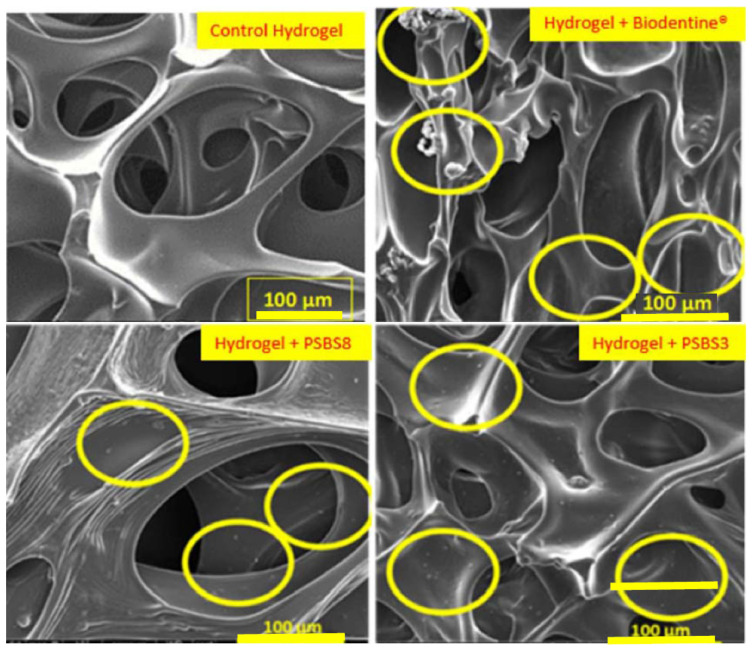
SEM images of functionalized hydrogels versus control hydrogels (scale bars = 100 µm). Yellow circles highlight particles present on the hydrogel surface [99].

The oral cavity contains a complex network of hard and soft tissues, each possessing specialized structural and functional characteristics essential for mastication, speech, esthetics, and general health [97]. Damage to these tissues, whether due to trauma, infection, inflammation, congenital anomalies, or neoplasms, can impair oral function and significantly impact quality of life. Therefore, regenerative strategies in dentistry must be tailored to the unique biological properties, healing potential, and biomechanical requirements of each tissue type [98]. The in vitro response of human gingival fibroblasts to a hydrogel functionalized with aluminum-free borosilicate glass was assessed. Two borosilicate glass particle types were tested, with Biodentine^®^ as a reference. The poly(L-lysine) dendrimer (DGL)-based hydrogel, with or without borosilicate particles, was characterized by µCT and SEM-EDX (Figure 4). Cytocompatibility and colonization assays over 7 and 14 days showed that adding borosilicate particles did not alter hydrogel porosity, while EDX confirmed their surface presence. Borosilicate-functionalized hydrogels enhanced cell growth, colonization, and calcium deposition, indicating promise for dental tissue regeneration [99].

### 3.1. Gingival Tissue

The gingiva is part of the masticatory mucosa, consisting of a keratinized epithelium supported by dense connective tissue. This provides a protective barrier against microbial invasion and mechanical trauma, while maintaining the integrity of the underlying periodontal structures [100].

Gingival defects can result from periodontal disease, trauma, or surgical resection [101]. While minor lesions may heal by natural epithelial renewal, large or deep defects require regenerative approaches to restore both structural integrity and esthetic contour. Key regenerative goals include reestablishing the epithelial barrier, integrating with adjacent tissues, and ensuring adequate vascularization.

**Figure 5:**
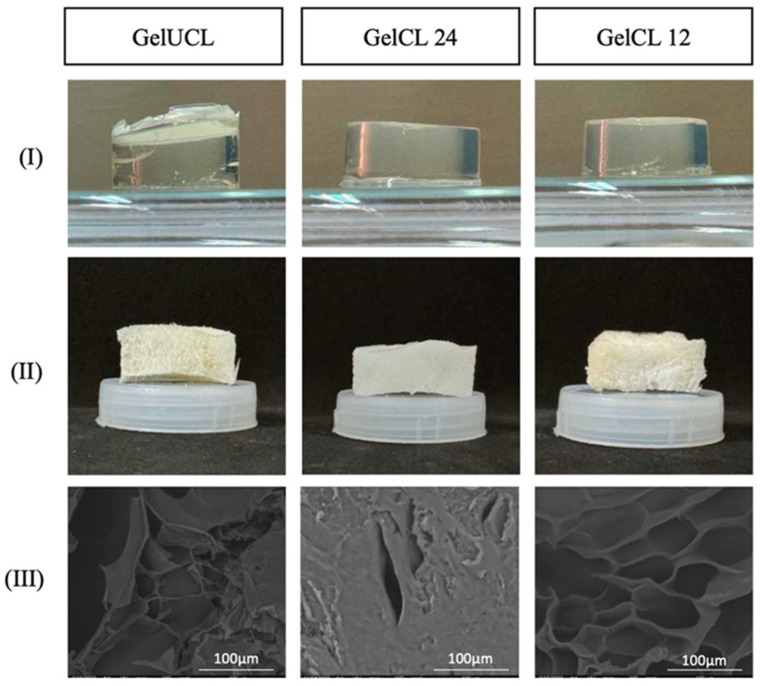
Morphological studies of gelatin hydrogels: GelUCL, GelCL24, and GelCL12. (**I**) Hydrogels in their fresh state. (**II**) Hydrogels after lyophilization. (**III**) Scanning Electron Microscopy (SEM) images of the lyophilized hydrogels [103].

Gelatin hydrogels show considerable potential in treating gingival recession due to their biocompatibility, tunable properties, and structural resemblance to the ECM [102]. Notably, gelatin naturally includes the arginine–glycine–aspartic acid (RGD) sequence, which facilitates cell adhesion and interaction. In a recent study, researchers investigated the physical and biological performance of crosslinked gelatin hydrogels using two different concentrations of a crosslinking agent (referred to as GelCL12 and GelCL24), compared to an uncrosslinked gelatin hydrogel. The crosslinking approach using EDC and NHS as coupling agents modifies the chemical structure of gelatin by forming amide bonds. This structural modification enhances the stability of the hydrogels while maintaining their biocompatibility and functional suitability as scaffolds. Experimental observations showed that GelCL12, characterized by a higher degree of crosslinking, outperformed both GelCL24 and GelUCL. Furthermore, GelCL12 exhibited a well-defined porous architecture with the largest pore diameter (83 µm), while GelCL24 exhibited the smallest pores. Overall, the concentration of EDC and NHS in the hydrogel matrix plays a crucial role in regulating cell proliferation. Regarding the crosslinking density, we can conclude that appropriately balanced concentrations improve hydrogel stability, resistance to degradation, and cell viability, while excessive levels may impair cell adhesion and proliferation. The findings demonstrated that both hydrogel formulations supported key cellular behaviors—such as proliferation, migration, and collagen type I (COL1) expression—of human gingival fibroblasts (HGF), highlighting their suitability as scaffolds for gingival tissue regeneration (Figure 5) [103].

**Figure 6:**
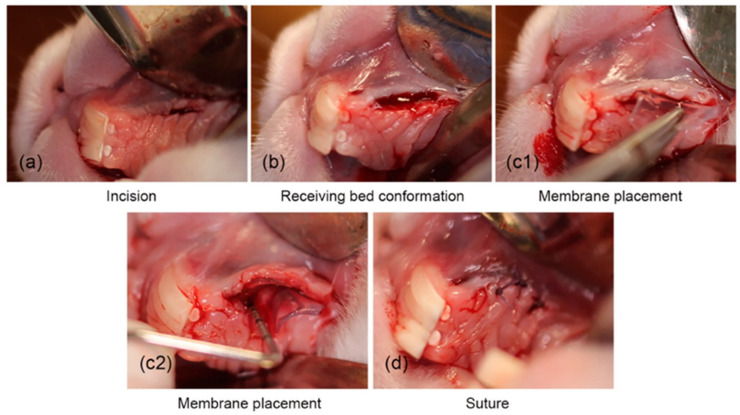
The surgical method for placing the cellulose hydrogel film includes the following steps: (**a**) making the surgical incision, (**b**) preparing the surgical site, (**c1**,**c2**) positioning the hydrogel film, and (**d**) suturing the incision [104].

**Figure 7:**
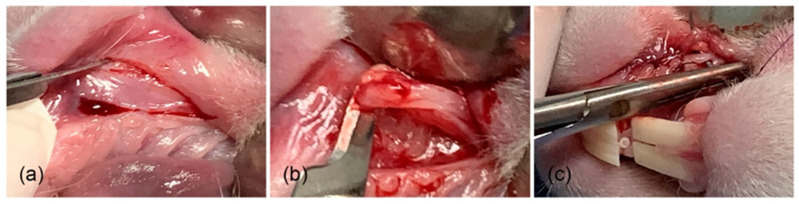
Surgical technique for membrane sampling: (**a**) preparation of the surgical site, (**b**) tissue sample extraction, and (**c**) suturing [104].

Cellulose hydrogels derived from agave bagasse were developed into membrane-like forms to support the regeneration of gingival connective tissue in lagomorphs [104]. An in vivo study was carried out using 16 New Zealand rabbits, with three treatment approaches applied. The biomaterials were randomly implanted in the gingival diastema region of the animals (Figure 6). Over a 16-week observation period, results indicated that the hydrogels played an active role in promoting oral connective tissue healing (Figure 7). The implanted materials were well-tolerated by the surrounding tissue, which exhibited a positive biological response.

### 3.2. Periodontal Ligament (PDL)

The PDL is a highly specialized fibrous connective tissue that anchors the tooth root to the alveolar bone via Sharpey’s fibers. It also functions as a shock absorber during mastication and plays a vital role in the mechanosensitivity and homeostatic maintenance of the adjacent bone and cementum [105]. Periodontal disease and trauma can lead to irreversible destruction of the PDL. Regeneration requires restoration of its unique hierarchical architecture, orientation of collagen fibers, and re-establishment of cementum–PDL–bone continuity. Functional regeneration is particularly challenging due to the need for both structural and biomechanical integration [106].

### 3.3. Alveolar Bone

The alveolar bone supports and houses the tooth roots. It undergoes continuous remodeling in response to mechanical loading, occlusal forces, and periodontal health. Bone loss can occur due to periodontitis, trauma, tumor resection, or tooth extraction. Regenerative requirements include restoration of bone volume, density, and architecture to support tooth retention or implant placement. Successful regeneration also requires adequate vascularization and integration with native bones [107].

### 3.4. Oral Mucosa

The oral mucosa lines the oral cavity and is composed of stratified squamous epithelium supported by a layer of vascular connective tissue. Depending on the region, it may be keratinized (masticatory mucosa) or nonkeratinized (lining mucosa). It serves as a barrier against physical, chemical, and microbial aggression, while contributing to sensation and lubrication. Loss of oral mucosa due to trauma, burns, surgical resection, or chronic ulcers require rapid epithelial resurfacing to restore barrier function. Regeneration must restore tissue flexibility, elasticity, and appropriate keratinization patterns according to the anatomical site [108].

### 3.5. Dental Pulp

The dental pulp is a soft connective tissue within the tooth, containing blood vessels, nerves, and odontoblasts. It is responsible for dentin formation, nutrient supply, and sensory function. Infection or trauma can cause irreversible pulpitis or pulp necrosis. Pulp regeneration aims to restore vascularization, innervation, and the odontogenic potential of resident cells to allow continued dentin formation. This requires biomaterials and growth factors that support angiogenesis, neurogenesis, and stem cell differentiation [109].

### 3.6. Dentin

Dentin is a mineralized tissue underlying enamel and cementum, composed primarily of hydroxyapatite crystals embedded in a collagen matrix. It is produced throughout life by odontoblasts, providing structural support and protecting the pulp. Dentin does not regenerate extensively after severe injury, instead relying on tertiary dentin formation by surviving odontoblasts or progenitor cells. Regenerative approaches focus on stimulating odontogenic differentiation and controlled mineral deposition to restore the protective and functional properties of dentin [110].

Table 4 outlines the principal features of oral tissues together with their regenerative requirements, addressing tissue type, primary function, major deteriorative factors, and regeneration-related aspects.

## 4. Applications of NHGs in Oral Tissue Engineering

NHGs, highly hydrated porous structure facilitates nutrient diffusion, cellular infiltration and incorporation of bioactive molecules, making them suitable for a wide range of regenerative applications in dentistry.

### 4.1. Periodontal Regeneration

**Figure 8:**
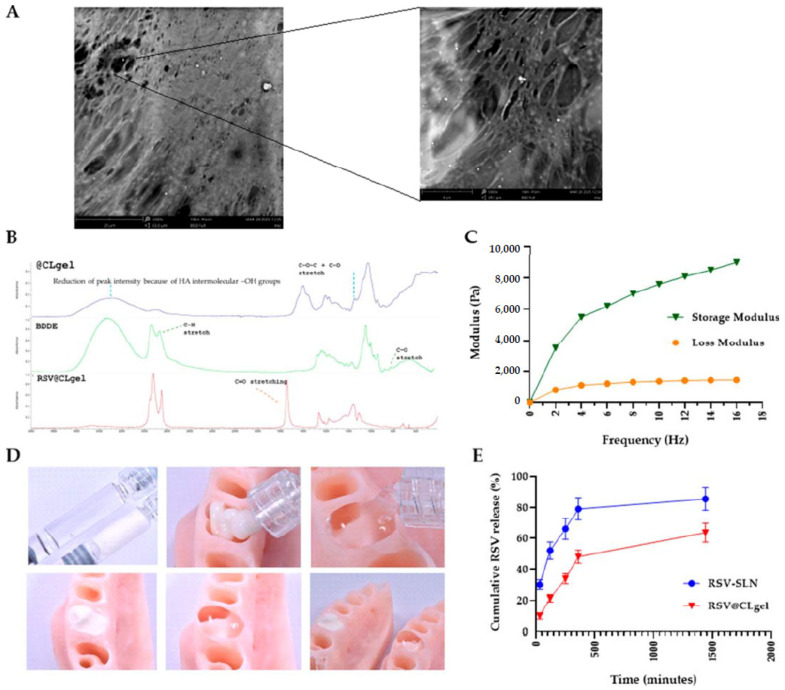
Physicochemical characteristics of the optimized RSV@CLgel. (**A**) SEM micrographs at varying magnifications. (**B**) FTIR spectra of RSV@CLgel. (**C**) Rheological properties of the hydrogel. (**D**) Shear-thinning behavior. (**E**) Comparative release profiles of RSV from RSV-SLN and RSV@CLgel [117].

Periodontitis is a chronic inflammatory condition and a major contributing factor to tooth loss in humans [111]. If left untreated, this inflammatory disease can cause irreversible damage to the supporting tissues of the teeth, leading to loosening or permanent loss of teeth, which can affect facial appearance, eating, and speech [112]. Although current treatments, such as surgery, root canals, curettage, and local antibiotic injections, help control symptoms and slow disease progression, they cannot completely restore the original structure and function due to the complexity of the periodontal pockets and supporting tissues [113]. NHGs provide a three-dimensional scaffold suitable for the regeneration of PDL, cementum, and supporting alveolar bone. In addition, hydrogels loaded with periodontal ligament stem cells (PDLSCs) or growth factors, such as platelet-derived growth factor (PDGF) and bone morphogenetic proteins (BMPs), demonstrated improved collagen fiber orientation and improved cement–PDL–bone interface formation. Also, chitosan and collagen-based hydrogels, or methacryloyl gelatin (GelMA) in particular, have been used to promote cell proliferation, ECM deposition and controlled release of bioactive molecules to support functional bone and periodontal regeneration [114,115,116]. A novel drug delivery platform was developed by incorporating resveratrol-loaded solid lipid nanoparticles into a crosslinked hyaluronic acid hydrogel (RSV@CLgel). The formulation was evaluated under hyperglycemic and inflammatory conditions to assess its impact on macrophage polarization, cytokine production, oxidative stress, mitochondrial activity, and osteoblast differentiation. Findings revealed desirable physicochemical characteristics, controlled release behavior, and significant biological activity, supporting its potential application in periodontal treatment for individuals with diabetes (Figure 8) [117].

**Figure 9:**
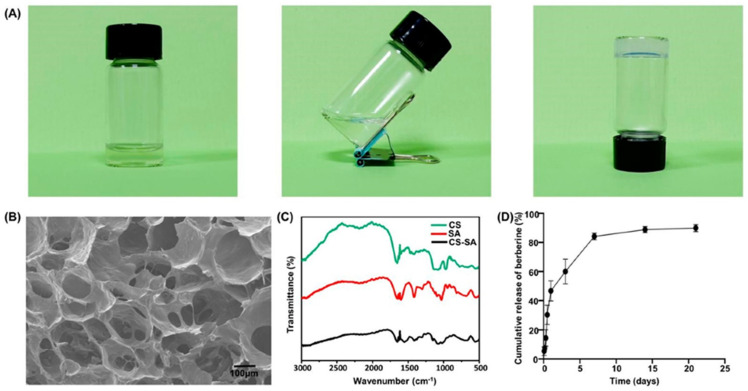
Characterization of the thermosensitive hydrogel: (**A**) Photograph showing the transformation of the CS/β–GP/SA-mixed solution into a hydrogel within 3 min at 37 °C. (**B**) SEM micrograph of the thermosensitive hydrogel structure. (**C**) FTIR spectra of the thermosensitive hydrogel. (**D**) Release profile of berberine from the thermosensitive hydrogel [118].

Because anatomical factors like root bifurcation and deep periodontal pockets can limit the effectiveness of basic periodontal treatment, topical medications are often used to enhance chronic periodontitis management. A thermosensitive hydrogel loaded with berberine that was developed to significantly reduce inflammation of periodontal tissue has shown anti-inflammatory and osteogenic effects by modulating the phosphatidylinositol-3-kinase/Protein Kinase B signaling pathway, as well as reducing protein levels (Figure 9) [118].

**Figure 10:**
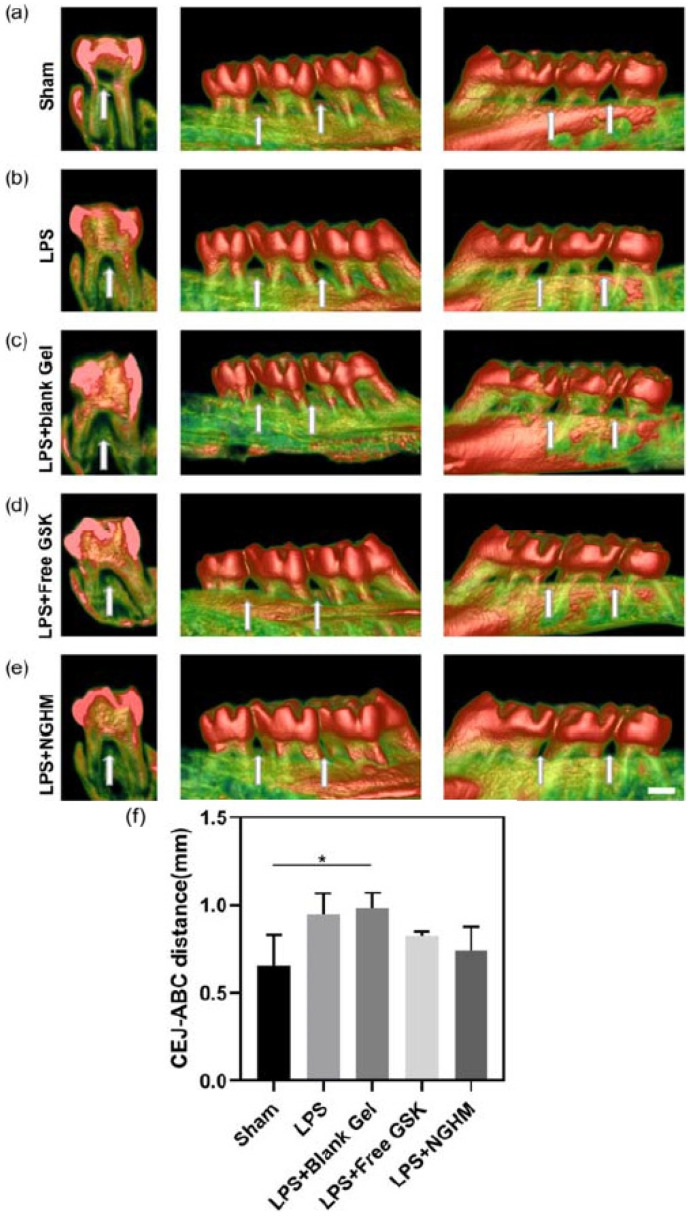
Reconstruction of maxillary alveolar bone in SD rat groups: sham (**a**), LPS (**b**), LPS + blank Gel (**c**), LPS + FreeGSK (**d**), and LPS + NGHM (**e**), with arrows pointing at the alveolar bone between the first and second molars and between the second and third molars. (**f**) Measurement of the CEJ–ABC distance using ImageJ 1.53p software. * *p* < 0.05. Scale bar: 1 mm [119].

A novel formulation of a collagenase-sensitive hydrogel was developed as a local delivery system for a nanoparticle-based nanodrug (NanoGSK) to inhibit endoplasmic reticulum stress and treat periodontitis. The results of drug release tests indicated remarkable drug release profiles, with enzymatic action in the local microenvironment of periodontitis. Overall, the hydrogel had a remarkable effect on inhibiting inflammation as well as alveolar bone regeneration (Figure 10) [119].

### 4.2. Bone and Alveolar Ridge Regeneration

Alveolar bone defects from oral trauma, congenital malformations, fenestration, or periodontal disease can greatly impair oral function [120,121].

Although bone grafts and substitutes have seen clinical success, achieving optimal alveolar bone regeneration remains challenging due to the oral cavity’s complex environment and specialized functions [122].

Hydrogels are materials that can be designed to enhance bone regeneration capacity and improve the efficiency of procedures in alveolar bone repair, including dental implant therapies [123,124].

In alveolar bone regeneration, NHGs can act as carriers for osteoinductive signals and progenitor cells, facilitating new bone formation in defects caused by trauma, periodontal disease, or tooth extraction [125,126,127]. Alginate and gelatin hydrogels enriched with calcium phosphate nanoparticles or bone morphogenetic protein 2 have shown promising results in promoting osteogenesis and mineralized tissue deposition. Injectable and/or osteoinductive growth factor-loaded hydrogel systems allow for minimally invasive administration into irregular bone defects and can be combined with particulate bone grafts to enhance stability and vascular infiltration, which is essential for successful alveolar ridge augmentation prior to dental implant placement [128].

**Figure 11:**
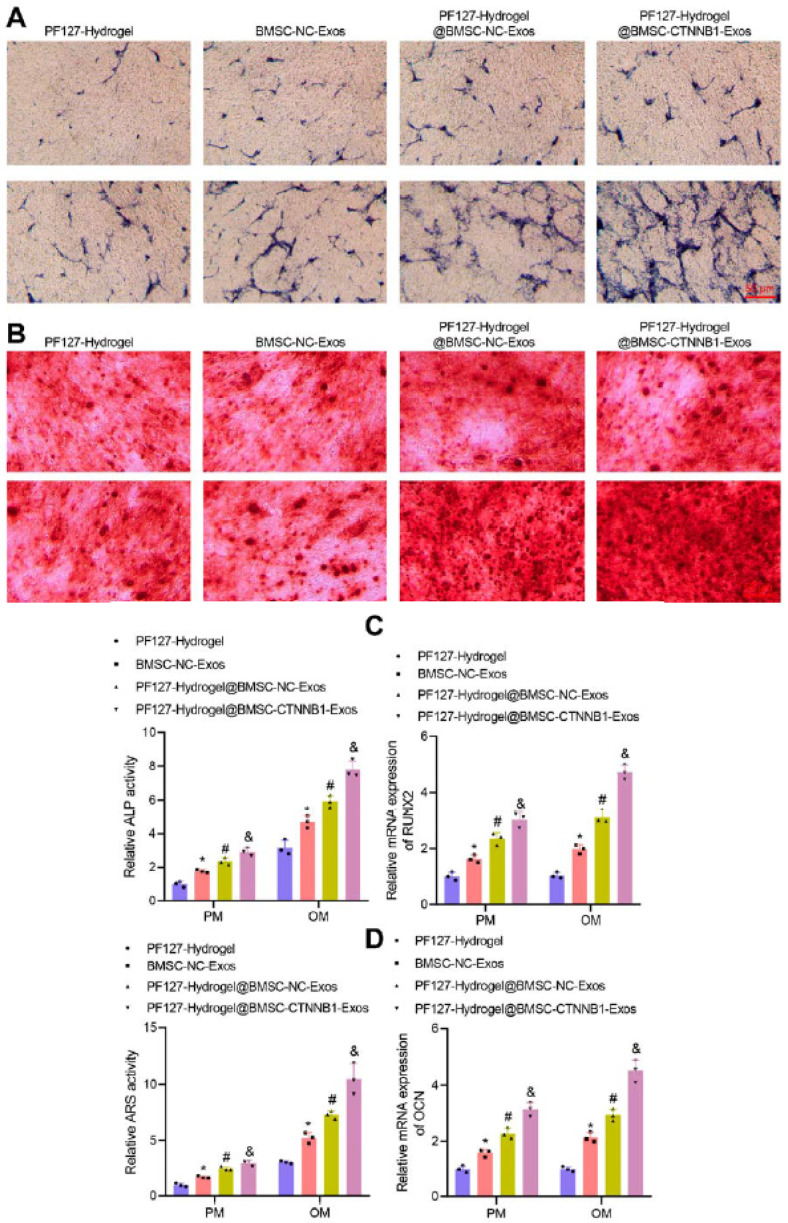
CTNNB1 enhances osteogenic differentiation of BMSCs via the miR-146a-5p/IRAK1/TRAF6 pathway. (**A**) ALP staining and quantification. (**B**) Alizarin red staining and quantification. (**C**) RUNX2 and (**D**) OCN mRNA levels by RT-qPCR. * *p* < 0.05 vs. oe-NC + inhibitor NC + sh-NC; # *p* < 0.05 vs. oe-CTNNB1 + inhibitor NC + sh-NC; & *p* < 0.05 vs. oe-CTNNB1 + miR-146a-5p inhibitor + sh-NC; *n* = 3 [130].

**Figure 12:**
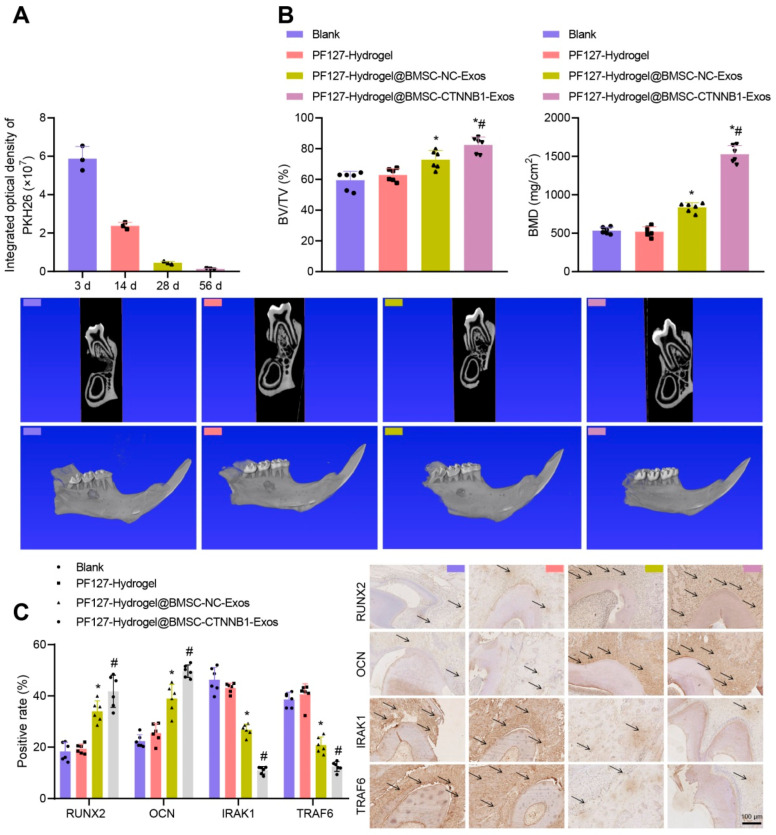
PF127 hydrogel loaded with BMSC-CTNNB1-Exos promotes alveolar bone regeneration in rats. (**A**) TPEF imaging and quantification of PKH26-labeled exosome distribution (*n* = 3). (**B**) Micro-CT analysis of alveolar bone defects (red box) and quantification (*n* = 6). (**C**) IHC staining for RUNX2, OCN, IRAK1, and TRAF6; black arrows indicate positive expression (*n* = 6). * *p* < 0.05 vs. PF127 hydrogel; # *p* < 0.05 vs. PF127 hydrogel@BMSC-NC-Exos [130].

Intelligent biomaterials and various engineered constructs have been designed to improve dental and periodontal regeneration, such as a three-layered nanocomposite hydrogel scaffold based on biopolymers, ceramics, growth factors and protein. The first layer or alveolar bone phase contains chitin-PLGA/nanobioactive glass ceramic (nBGC)/platelet-rich plasma-derived growth factors. The second layer or PDL phase is composed of chitin-PLGA/fibroblast growth factor. The third layer, the cemented phase, is composed of chitin-PLGA/nBGC/cement protein 1. The results of the in vivo experiments showed that the hydrogel scaffold implanted in rabbits promoted complete healing of defects and the formation of well-defined new alveolar bone tissue [129]. Pluronic F127 hydrogel is a promising material for bone regeneration, although its molecular mechanisms are not fully understood. This temperature-sensitive hydrogel, loaded with exosomes derived from osteocyte mesenchymal stem cells and enriched with a key osteogenic gene, was tested to repair alveolar bone defects. Results of in vivo tests showed that the hydrogel promoted new bone formation. The enriched hydrogel was also able to stimulate osteogenesis (Figure 11) and support alveolar bone regeneration in rats (Figure 12) [130].

### 4.3. Soft Tissue Repair (Gingiva and Oral Mucosa)

Hydrogels play a crucial role in soft tissue engineering due to their moisture retention properties, which support the proliferation of keratinocytes and fibroblasts [131]. Collagen and hyaluronic acid hydrogels have been used to repair gingival recession defects and accelerate the healing of oral mucosal lesions [132,133].

They can be designed to match the mechanical flexibility of soft tissues, thereby enhancing integration and reducing scar formation. In addition, hydrogels containing antimicrobial agents (e.g., polysaccharide gums, bioactive plant components) or essential oils provide infection control during the healing process of the oral mucosa [134,135,136].

Chitosan hydrogel is effective in treating gum and oral mucosal diseases, including canker sores [137].

Mouthwashes, toothpastes, lozenges and chewing gums containing chitosan and plant extracts provide antimicrobial properties that help combat oral biofilm and reduce the presence of Streptococcus mutans in the mouth [138,139]. The combination of chitosan complexes and fluoride improves fluoride absorption, providing better cavity protection. In addition, chitosan-based endodontic cements help reduce inflammation and promote bone regeneration [140,141].

### 4.4. Dental Pulp Regeneration

**Figure 13:**
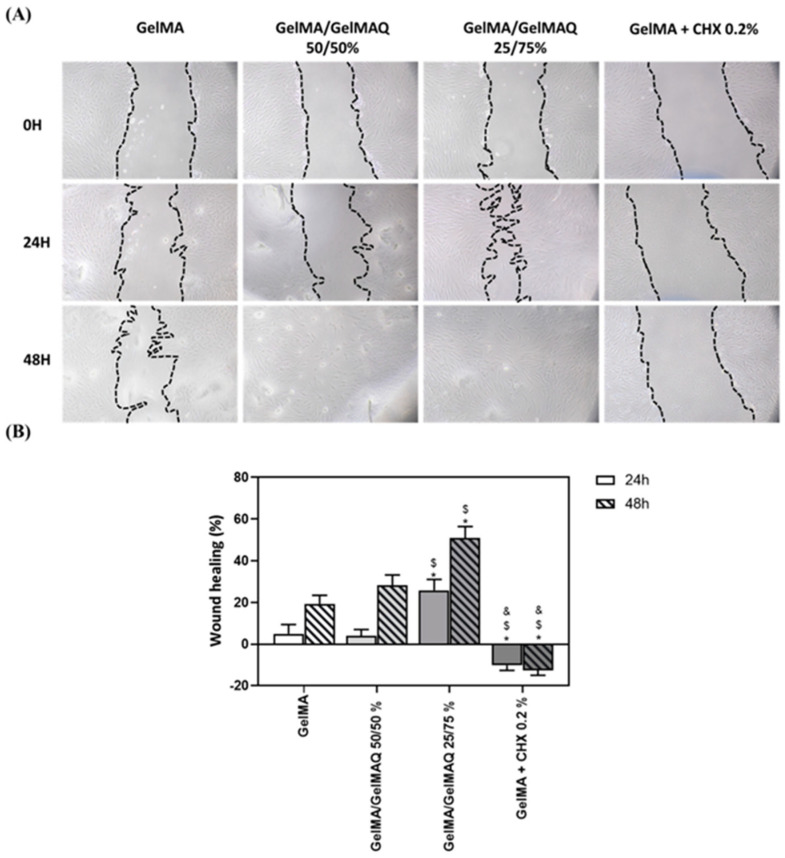
Wound healing assay on human gingival fibroblasts after treatment with various GelMA formulations. (**A**) Representative images at 24 h and 48 h (100×). (**B**) Percentage wound closure quantified by ImageJ, v1.51k (NIH, Bethesda, MD, USA), (mean ± SEM, *n* = 6). Statistical analysis: ANOVA with LSD post hoc; * *p* < 0.05 vs. GelMA, $ *p* < 0.05 vs. GelMA/GelMAQ 50/50%, & *p* < 0.05 vs. GelMA/GelMAQ 25/75% [146].

Hydrogel-based scaffolds have garnered significant attention due to their remarkable properties, including biocompatibility, safety, and cost-efficiency. Their ease of fabrication, ability to be tailored for different applications, and simplicity in synthesis make them highly versatile. Moreover, hydrogels facilitate effective cellular adhesion by closely mimicking natural extracellular matrix components [142]. These characteristics make hydrogels an ideal choice as delivery carriers for stem cells, such as human dental pulp stem cells (hDPSCs), in efforts to regenerate damaged dentin and pulp tissues [143]. For the regeneration of the pulp-dentin complex, hydrogels serve as injectable scaffolds capable of delivering dental pulp stem cells (DPSCs) and angiogenic/neurogenic growth factors, such as vascular endothelial growth factor (VEGF) and nerve growth factor (NGF) [144]. Gelatin methacrylate (GelMA) and collagen hydrogels have been shown to be particularly effective in supporting odontoblast differentiation and tubular dentin formation, while promoting revascularization and innervation in the pulp chamber [145]. GelMA hydrogels functionalized with quaternary ammonium groups (GelMAQ) showed enhanced antibacterial activity. The 25/75 GelMA/GelMAQ blend demonstrated strong antibacterial effects, high biocompatibility, and regenerative potential, supporting its use in periodontal tissue regeneration (Figure 13) [146].

The injectability of certain hydrogel formulations allows them to fill complex pulp spaces and adapt to irregular root canal geometries [147,148].

Fibrinogen-blood hydrogels (with different concentrations) were studied for regenerative endodontic treatment of immature necrotic teeth. Evaluation of biomimetic properties, ECM formation and angiogenic potential showed the formation of neovessel-like structures, similar to that of human dental pulp [149].

### 4.5. Drug and Growth Factor Delivery Systems

NHGs provide an excellent platform for localized and sustained release of therapeutic agents in the oral environment, where systemic administration is often ineffective due to rapid clearance [150,151]. Their porous, crosslinked structure allows the encapsulation of antibiotics, anti-inflammatory agents, growth factors, and even gene therapy vectors [152].

Numerous formulations of hydrogels in the form of gels, films or tablets have been developed, including commercially, as carriers of various drugs. For example, a commercial hydrogel based on hydroxypropylmethylcellulose (HPMC) for the delivery of benzocaine to the buccal area demonstrated the absence of oral mucosal pain for 4–6 h [153]. pH-sensitive hydrogel films containing cross-linked sodium tripolyphosphate, a ternary mixture of chitosan, guar gum, and polyvinylpyrrolidone were formulated for oral administration of ciprofloxacin hydrochloride as a drug dissolved in an aqueous gel solution. Test results indicated that the hydrogel exhibited a pH-dependent response, with 30% of the drug being released within the first 30 min in simulated gastric fluid. In parallel, sustained drug release was observed in simulated intestinal fluid and phosphate-buffered saline [154]. The novel formulation of thermosensitive hydrogels incorporating doxorubicin and gold nanoparticles has demonstrated superior tumor ablation. The photothermal capabilities of the gold nanoparticles enhance localized heating, enabling targeted drug release and significantly boosting therapeutic effectiveness [155].

### 4.6. Wound Healing and Anti-Inflammatory Applications

Hydrogels create a moist wound environment that promotes epithelial migration, angiogenesis and ECM remodeling. NHGs enriched with bioactive agents—such as curcumin, aloe vera or essential oils—exhibit enhanced antimicrobial and anti-inflammatory properties, making them suitable for post-surgical oral wounds, extraction sockets, and traumatic injuries [156,157,158,159,160,161].

Their ability to modulate inflammatory responses helps prevent excessive tissue degradation while supporting regeneration, especially in chronic inflammatory conditions such as periodontitis or oral lichen planus.

The summary of applications of NHGs in oral tissue engineering presented in Table 5 is organized according to the specific field of application, types of hydrogels used and their corresponding functional roles.

## 5. Challenges and Limitations

Although NHGs hold promises for multifunctional scaffolds that combine structural support, bioactivity, and antimicrobial protection, several challenges hinder their widespread clinical adoption. These limitations range from material performance to translational and regulatory hurdles and must be addressed to ensure safe and effective application in oral tissue engineering.

### 5.1. Mechanical Deficiencies

NHGs, due to their high water content and polymeric structure, often exhibit low tensile strength and poor load-bearing capacity. This mechanical fragility becomes a significant limitation in oral applications, where the scaffolds may be exposed to dynamic forces from mastication, tongue movement, and speech. Incorporation of EOs or other bioactive compounds can further modify the microstructure of the hydrogel, sometimes soften the matrix or disrupt crosslinking, leading to reduced mechanical stability. Although reinforcement strategies exist (e.g., blending with nanofibers, use of composite formulations), achieving an optimal balance between mechanical robustness and the desired drug release profile remains a challenge.

Reinforcement (mechanical, compositional, and structural) strategies that show good promise specifically for alveolar bone or craniofacial bone/hard tissue regeneration are the use of (i) inorganic fillers; (ii) interpenetrating polymer networks, often one rigid, one flexible, so that the networks dissipate energy and resist deformation; (iii) crosslinkers that produce stronger bonds (vinyl/methacrylate/acrylate groups); (iv) scaffolds or fiber networks [162].

### 5.2. Immunogenicity and Control of Biodegradation Rate

Even though NHGs are generally biocompatible, immunogenic responses can still occur, especially if the starting material contains residual proteins, endotoxins, or plant-derived impurities. EOs, despite their natural origin, can cause hypersensitivity reactions or local irritation in certain individuals, especially at higher concentrations.

Furthermore, the biodegradation rate of the hydrogel must match the rate of tissue regeneration, a mismatch can lead to premature loss of the scaffold or prolonged presence of foreign bodies. Depending on their chemical composition, bioactive components can accelerate or delay degradation by interacting with the polymer network of the hydrogel or by modifying its hydrophilicity. Controlling this degradation profile in vivo is difficult, especially in the enzymatically rich and microbiologically active oral environment.

### 5.3. Scaling up and Clinical Translation

Most studies on NHGs enriched with bioactive agents or other natural molecules are still in the laboratory or preclinical stages, with limited data on large-scale manufacturing. Scale-up of production requires a reproducible supply of raw materials, both biopolymers and other biocomponents, while maintaining constant chemical composition and bioactivity. There may be batch-to-batch variability in purity, composition and mechanical properties, including due to differences in plant origin, harvesting conditions and extraction methods, affecting reproducibility. Also, sensitivity to sterilization can be an issue, as some hydrogels can lose their bioactivity or structure when sterilized on an industrial scale. Also, complex extraction or purification processes hinder mass production.

Regarding preclinical validation, on the one hand, the most frequently reported in vitro studies test cytotoxicity, cell adhesion, proliferation and differentiation using oral cell lines (e.g., gingival fibroblasts, periodontal ligament cells). On the other hand, in vivo models (often mice, rats, rabbits or dogs) evaluate the performance of the hydrogel in the regeneration of periodontal tissues, oral mucosa or bone defects. A particularly important category is represented by long-term studies, which evaluate immunogenic responses and functional integration of the hydrogel with native tissue. These are, unfortunately, less frequently reported.

From a clinical point of view, functional outcomes such as tissue regeneration, vascularization and restoration of oral functions are critical indicators in assessing success.

The translation of natural hydrogel-based materials, particularly in overcoming regulatory barriers and achieving standardization, should primarily focus on bridging the gap between promising laboratory discoveries and their clinical implementation, namely through standardized manufacturing, characterization, and validation protocols.

Regulatory barriers remain a critical challenge, however, as NHGs often face scrutiny due to their biological variability, potential immunogenicity, and batch-to-batch inconsistencies. Ensuring material standardization, including reproducibility in composition, mechanical properties, and degradation profiles, is essential to meet regulatory requirements and build clinical confidence.

Streamlined preclinical testing, harmonized quality control guidelines, and early regulatory engagement will be essential to accelerate the safe and effective translation of NHGs into oral healthcare solutions.

### 5.4. Regulatory and Ethical Considerations

Regulatory approval for NHGs is complicated by the dual nature of the product: it acts as both a medical device (scaffold) and a drug (bioactive delivery system). This combination often triggers more stringent regulatory pathways that require comprehensive toxicological, pharmacokinetic, and efficacy data. Bioactive agents must meet rigorous standards of safety and purity when applied to biomedical scaffolds. Ethical considerations also arise when sourcing natural materials, particularly if plant harvesting impacts biodiversity or involves unsustainable practices. Last but not least, there may be regulatory hurdles due to the biological origin that raise issues related to immunogenicity, pathogen transmission, and safety. In addition, the patient consent procedure should clearly communicate potential risks, including allergic reactions or unforeseen drug interactions.

An overview of the main constraints of NHGs in oral tissue engineering, along with their associated impact is summarized in Table 6.

## 6. Future Directions—Overcoming Challenges for NHGs in Oral Tissue Engineering

As next-generation materials, NHGs can address several objectives, particularly the following:

(i) Improving mechanical strength to withstand masticatory forces through composite or cross-linked hydrogel systems.
(ii) Optimizing degradation rates to align with oral wound healing time and tissue regeneration.
(iii) Improving antimicrobial properties to resist the oral microbiome and prevent infection.
(iv) Standardizing bio fabrication for consistent and scalable production suitable for intraoral applications.
(v) Incorporating bioactive molecules (e.g., enamel matrix proteins, salivary peptides) to promote site-specific cellular differentiation.
(vi) 3D bioprinting of patient-specific scaffolds for precise anatomical fit to complex oral defects.
(vii) Using low-temperature or non-destructive sterilization methods to preserve the bioactivity of NHGs.

## Figures and Tables

**Table 1:** Comparative benefits and drawbacks of natural versus synthetic hydrogels for dental use.

Natural hydrogels	**Advantages**	**Disadvantages**	**Applications**
	Biocompatibility	Poor mechanical properties	Wound healing (collagen, fibrin, chitin, chitosan, HA, pullulan)
	Biodegradability	High water content	Wound dressing (albumin, silk fibroin, wheat gluten, alginate, cellulose)
	Typically, inexpensive	Source-induced batch variability	Drug delivery (gelatin, albumin, sericin, soy protein isolate, chitosan, alginate, carrageenan, cellulose, starch, dextran, pullulan)
	Derived from substances found within the ECM in vivo	Poor stability over a long period of time	3D scaffolds (silk fibroin, gelatin, soy protein isolate, dextran, xanthan gum, cellulose, chitin
	Can be modified to include binding sites or alter stiffness	May lack reproducibility	
	Promotes cell adhesion, proliferation and growth	Variable solubility in water Sensitive to environment and pH	
Synthetic hydrogels	Customizable composition	Large excess of water	Wound Healing (as dressings)
	Modifiable stiffness	May have cytotoxic effect	Drug delivery systems: -Targeted release (encapsulated antibacterial agents, fluoride, or other therapeutic compounds) for localized treatment of oral infections, caries, and periodontitis -Controlled release (to release drugs slowly and in response to stimuli, such as pH or temperature changes)
	High Reproducibility	Requires addition of binding sites to allow cells to adhere	Tissue scaffolds in periodontal or dental pulp regeneration
	High durability, Better processability, and tunable characteristics	Lower cytocompatibility, can be biologically inert, more costly to produce, and may pose toxicity risks if they release harmful byproducts upon degradation.	-Orthodontic tooth movement regulation -Enamel and dentin remineralization

**Table 2:** Overview of strengths and weaknesses of naturally derived hydrogels.

Hydrogel	Advantages	Constraints
Collagen	Excellent biocompatibility; mimics natural ECM; supports cell adhesion and proliferation	Weak mechanical strength; fast degradation; immunogenicity (bovine sources)
Gelatin	Thermo-responsive; cost-effective; easy to modify	Poor mechanical properties; enzymatic degradation in vivo
Chitosan	Antibacterial; hemostatic; promotes osteogenesis	Limited solubility at physiological pH; low elasticity
Alginate	Easy gelation (ionic crosslinking); non-immunogenic	Poor cell adhesion; brittle mechanical properties
Hyaluronic Acid (HA)	Promotes cell migration and angiogenesis; highly hydrophilic	Rapid degradation; weak mechanical strength
Fibrin	Autologous source; supports angiogenesis and cell migration	Fast degradation; poor mechanical integrity

**Table 3:** Advantages and disadvantages of natural, synthetic, and hybrid hydrogel systems with respect to composition, biocompatibility, mechanical stability, degradation, and functionalization.

Hydrogels	Composition and Origin	Biocompatibility and Bioactivity	Mechanical Properties and Stability	Degradation and Biodegradability	Processability and Functionalization
Natural	Derived from biological sources such as proteins (e.g., gelatin, collagen) or polysaccharides (e.g., alginate, chitosan, hyaluronic acid)	-Highly biocompatible -Intrinsic bioactivity (e.g., cell adhesion, enzymatic degradation)	-Poor mechanical strength and sensitivity to environmental conditions (e.g., temperature, pH)	Degradable through enzymatic or hydrolytic pathways	-Limited processability -Functionalization is possible but complex
Synthetic	Engineered from polymers like polyethylene glycol (PEG), polyvinyl alcohol (PVA), or polyacrylamide	-Generally inert -Non-immunogenic	-Tunable and superior mechanical properties, -Durability, -Elasticity, -Responsiveness	-Controllable degradation -Non-degradable, toxic residues	-Highly processable -Precise functionalization for drug delivery, sensing, or mechanical tuning.
Hybrid	Blend natural and synthetic polymers to combine the bioactivity of natural materials with the tunability of synthetic ones	Balance between natural components (bioactivity), and synthetic (stability and strength)	-Improved mechanical performance -Biofunctionality	Enable tunable degradation rates	Ease engineered for specific applications

**Table 4:** Characteristics of oral tissues and their regenerative requirements.

Tissue Type	Main Function	Major Causes of Damage	Regeneration Focus
Gingival tissue	Barrier, esthetics	Periodontitis, trauma, surgery	Epithelial barrier, vascularization, contour
Periodontal ligament	Tooth anchorage, shock absorption	Periodontitis, trauma	Fiber orientation, cementum-PDL-bone interface
Alveolar bone	Tooth support	Periodontitis, trauma, extraction	Bone volume, density, vascularization
Oral mucosa	Protective lining	Burns, ulcers, surgery	Rapid coverage, elasticity, keratinization
Dental pulp	Sensory, dentinogenesis	Caries, trauma	Angiogenesis, neurogenesis, odontogenesis
Dentin	Structural support	Caries, abrasion, fracture	Odontoblast stimulation, mineral deposition

**Table 5:** Summary of applications of NHGs in oral tissue engineering.

Application Area	Common Hydrogel Types	Functional Role
Periodontal regeneration	Collagen, chitosan, alginate	Scaffold for PDLSCs, growth factor delivery, fiber orientation
Bone/alveolar ridge regeneration	Alginate, gelatin, collagen	Osteoconduction, osteoinduction, injectable defect filling
Soft tissue repair	Collagen, hyaluronic acid	Moisture retention, fibroblast/keratinocyte proliferation
Dental pulp regeneration	Collagen, GelMA	DPSC delivery, angiogenesis, odontogenesis
Drug/growth factor delivery	Chitosan, gelatin, alginate	Sustained local release, targeted therapy
Wound healing, antiinflammatory	Chitosan, Aloe Vera gel, alginate	Moist healing, infection control, inflammation modulation

**Table 6:** Main challenges of NHGs in oral tissue engineering.

Challenge	Primary factor	Impact on Application
Mechanical weaknesses	Low tensile or compressive strength; bioactive compounds -induced matrix softening	Limits used in load-bearing oral sites
Immunogenicity and biodegradation control	Allergic reactions; unpredictable degradation in vivo	Risk of inflammation, scaffold mismatch
Scaling up and translation	Raw material variability; stability issues	Hinders reproducibility and market readiness
Regulatory and ethical barriers	Device–drug combination regulations; sustainability concerns	Slows approval, raises compliance demands

## Data Availability

No new data were created or analyzed in this study.
